# Towards the use of mixed methods inquiry as best practice in health outcomes research

**DOI:** 10.1186/s41687-018-0043-8

**Published:** 2018-04-11

**Authors:** Antoine Regnault, Tom Willgoss, Skye Barbic, On behalf of the International Society for Quality of Life Research (ISOQOL) Mixed Methods Special Interest Group (SIG)

**Affiliations:** 1 Modus Outcomes, 61, Cours de la Liberté, 69003 Lyon, France; 2 Roche Products Ltd., Welwyn Garden City, UK; 3 The University of British Columbia, Vancouver, Canada

## Abstract

Mixed methods research (MMR) has found an increased interest in the field of health outcomes research. Consideration for both qualitative and quantitative perspectives has become key to contextualising patient experiences in a clinically meaningful measurement framework. The purpose of this paper is to outline a process for incorporating MMR in health outcomes research to guide stakeholders in their understanding of the essence of mixed methods inquiry. In addition, this paper will outline the benefits and challenges of MMR and describe the types of support needed for designing and conducting robust MMR measurement studies. MMR involves the application of a well-defined and pre-specified research design that articulates purposely and prospectively, qualitative and quantitative components to generate an integrated set of evidence addressing a single research question. Various methodological design options are possible depending on the research question. MMR designs allow a research question to be studied thoroughly from different perspectives. When applied, it allows the strengths of one approach to complement the restrictions of another. Among other applications, MMR can be used to enhance the creation of conceptual models and development of new instruments, to interpret the meaningfulness of outcomes in a clinical study from the patient perspective, and inform health care policy. Robust MMR requires research teams with experience in both qualitative and quantitative research. Moreover, a thorough understanding of the underlying principles of MMR is recommended at the point of study conception all the way through to implementation and knowledge dissemination. The framework outlined in this paper is designed to encourage health outcomes researchers to apply MMR to their research and to facilitate innovative, patient-centred methodological solutions to address the complex challenges of the field.

## Background

*“We need a moral and methodological community that honors and celebrates paradigm and methodological diversity.”* Denzin, [1]; (pp.425).

Mixed methods research (MMR) has been established for more than 50 years as a methodological approach in the social and behavioural sciences and is now well accepted and commonly used in health sciences [2–4]. In line with the call to “measure what matters” to patients, patient reported outcomes are increasingly being used in clinical care and research. However, a recent review of studies documenting the development of patient-reported outcomes (PRO) measures highlights that only 11% of PROs have been developed by actually asking patients which outcomes are important to them [5]. This highlights a clear application for MMR to combining qualitative and quantitative methods in health research to ensure a focus on patient-identified priorities, scientific rigour, and improved patient outcomes.

In 2012, a special section of *Quality of Life Research* was dedicated to applied MMR [6]. In this issue, MMR was described as an approach to inform the content validity of PROs within the early development phase. The Food and Drug Administration (FDA) also outlined a clear role for MMR in their roadmap to patient focused measurement [7]. Shortly after, a Special Interest Group (SIG) was created within the International Society for Quality of Life Research (ISOQOL) to promote the use of MMR and encourage health outcomes researchers to embrace the MMR paradigm. As a SIG, we believe that there is a need for guiding principles for researchers who wish to undertake MMR. With this position paper, we aim to provide a framework for MMR in health outcomes by outlining the characteristics of this methodology, what can be expected and where caution should be exercised.

## Defining features of mixed methods research

The application of the MMR paradigm in the health outcomes field can be rooted in the widely accepted definition by Tashakkori and Creswell: *“Mixed Methods Research is a research in which the investigator collects and analyses data, integrates the findings, and draws inferences using both qualitative and quantitative approaches or methods in a single study or program of inquiry”* [8] (pp.2).

In the spirit of pragmatism which underlies this methodology, we assert that utilizing MMR in the health outcomes field should not be limited to the application of a closed list of possible methodological options, but should be viewed as a framework characterized by three key defining features described in Table 1.

**Table 1:** Defining features of the mixed methods research framework

1. A specific research question is to be addressed using quantitative and qualitative components (data and/or methods)	
2. The quantitative and qualitative components are articulated purposely and prospectively in a well-defined, pre-specified research design	
3. The response to the research questions is supported by an integrated set of evidence generated from both the qualitative and quantitative component of the research (meta-inference)	

In this framework, not only should both qualitative and quantitative strands be used by the researcher, but they should be complemented in a relevant research design that is set a priori. They may address distinct, specific research questions but they contribute to the same overall end purpose of the MMR. Once the overall purpose, and the specific research questions of the qualitative and quantitative strands, are well-defined and procedures are outlined, a clear plan for interaction between the qualitative and quantitative research components is needed. This point relates to the notion of meta-inference that requires qualitative and quantitative evidence not be considered independently, but interpreted together as a single body of evidence [3]. Importantly, the importance of the specification relates to the research design, and especially the articulation of the qualitative and quantitative strands, but it obviously does not necessary apply within the research strands, as, in many instances, in particular for qualitative research, a full prespecification may not be appropriate, the research being of exploratory nature.

The characterization of MMR is driven neither by the data collection process, nor by the analysis technique. In MMR, it is not necessary that the qualitative and quantitative streams involve data collected with the same respondents. A well-designed MMR study may combine qualitative and quantitative data from different samples of individuals to address a single research question, combining rigorous qualitative and quantitative evidence. Conversely, the collection of qualitative and quantitative data for the same individuals does not necessarily allow for a proper MMR solution as it may be done to address different research questions or without considering both data sets in an integrated approach.

Many options are available to the health outcomes researcher looking to utilize MMR, depending on the research question and design. We assert that MMR should not be restricted to any specific research design or methodology, but rather the design that is best suited to answer the research question posed. The articulation of the qualitative and quantitative elements can be performed in various designs that are well described in the methodological literature (e.g. convergent, or parallel or concurrent designs; sequential designs; embedded designs) [4]. The choice of the appropriate design and analysis technique (qualitative and quantitative) remains the responsibility of the researcher who should be guided by the principles outlined above.

## Benefits and challenges of mixed methods research

### Benefits

MMR allows a research question to be studied from different perspectives. For example, one can combine the rich, subjective insights on complex realities from qualitative inquiry, with the standardized, generalizable data generated through quantitative research. When applied, MMR allows respective strengths and weaknesses of each approach to complement each other.

Since its conception in 2015, the Mixed Methods SIG of ISOQOL has identified and discussed many different applications of MMR in the health outcomes field: exploration of patient experiences to support the development of conceptual models with group concept mapping [9–11]; development of new clinical outcome assessment instruments with integrated qualitative and early quantitative analyses with Rasch model [12–15]; quantitatization of qualitative data to support conceptual saturation analyses [16, 17]; use of qualitative information to support the interpretation of quantitative patient-reported outcomes results [18, 19], to name but a few.

These examples show that MMR can help us to address common questions in the health outcomes field. This is typified by the inductive and iterative process characteristic of the development of new PROs. It also allows for flexibility and the ability to make the most of small samples. MMR enables a pragmatic path forward to conduct health outcomes measurement research in rare disease populations [20] or populations that are often difficult to recruit for research purposes (e.g., paediatrics, acute mental health, palliative). In a clinical research context, the versatility of MMR makes it a method of choice for hypothesis generation on PRO endpoints, especially in phase II trials.

Finally, a critical strength of MMR approaches is that they typically capitalize on data reflecting individual lived experiences (in the qualitative strand). This ensures that the results are considered from the patient-perspective. Incorporating the patient voice in MMR helps ensure that the research is focused on the needs and priorities of patients. Moreover, MMR can facilitate the involvement of other key stakeholders, such as partners, family members, and/or other knowledge users, in the process of developing the research question(s) and outlining the research designs. In this context, it appears clearly that MMR is a strong option to leverage effective patient engagement and support ongoing research focused on patient-identified priorities and the improvement of patient outcomes [21].

### Challenges

Despite some clear benefits, the application of MMR in the health outcomes research does not come without challenges. One major hurdle is that MMR is demanding in terms of methodological skillsets. MMR requires a team of researchers who are experienced in both qualitative and quantitative research, and in MMR designs. Indeed, as with traditional qualitative or quantitative methodologies, best practices should be applied rigorously across the multiple methods, but also in the way the quantitative and qualitative strands are articulated. A particularly critical issue in this context is that of meta-inference, in which the qualitative and quantitative strands connect. Meta-inference should be carefully specified, and researchers should be aware of the challenges of interpreting conflicting results.

The application of MMR can also raise practical considerations, particularly as the integration of both qualitative and quantitative data can require additional resources and time. However, it should be noted that this additional burden can often be offset against the potential benefits of MMR, particularly where multiple insights support the investigation of complex research questions or small populations.

Finally, we acknowledge that some theoretical debate still exists on how - or even whether - quantitative and qualitative paradigms can be mixed, a debate typified by the ‘paradigm wars’ of the second half of the twentieth century [1]. Such challenges stem from differences in the underlying ontological and epistemological positions of positivism (that a single objective reality exists) and constructivism (that reality is a subjective construct and therefore multiple realities exist). Even though the MMR paradigm goes beyond simply mixing the quantitative and qualitative paradigms and builds a third path, some purists continue to question this third paradigm, considering the very nature of the qualitative and quantitative paradigms irreconcilable. However, as Maxwell and Mittapalli argue [22], there is an alternative position to positivism and constructivism – critical realism. Critical realists deny that we have any objective or certain knowledge of the world, and accept the possibility of alternative valid accounts of any phenomenon. They argue that all theories about the world are grounded in a particular perspective and worldview, and all knowledge is partial, incomplete, and fallible. As such, critical realism provides a philosophical stance that is compatible with MMR in that it acknowledges the methodological characteristics of both qualitative and quantitative research, and can facilitate communication and cooperation between the two. Against the background of this ongoing debate, it is clear from the growing literature that the acceptance of MMR is increasing as the health outcomes research community continues to promote and celebrate methodological diversity.

## Conclusions and recommended reading

Two conditions appear critical for the continued development of credible and robust MMR. First, health outcomes researchers have the potential to learn about the different MMR methodologies and outline how MMR can be used to more thoroughly answer health outcomes research questions. This may include increasing knowledge about the underlying philosophy and history of MMR, examples of MMR research designs and principles, and the pros and cons of this approach above a purely qualitative or quantitative inquiry. To support this journey we provide a list of recommended texts which can form a starting point for the curious researcher.

Second, an open dialogue and collaboration between health outcome researchers with positivist or interpretivist leanings should be encouraged to prepare the ground for robust MMR. In this context, it will be possible to design health outcomes research studies in which the whole is greater than the sum of its parts and allow the research community to further the science through providing innovative solutions to our research challenges.

## Abbreviations

FDA: Food and Drug Administration
ISOQOL: International Society for Quality of Life Research
MMR: Mixed Methods Research
PRO: Patient-Reported Outcomes
SIG: Special Interest Group

## References

[1] Denzin Norman K. (2010). Moments, Mixed Methods, and Paradigm Dialogs. Qualitative Inquiry.

[2] Creswell JW, Designing PCVL (2007). Conducting mixed methods research.

[3] Tashakkori A, Teddlie C. Handbook of mixed Methods in social behavioral research. Thousand Oakc,CA: Sage Publications; 2003.*.

[4] Teddlie C, Tashakkori A (2009). Foundations of mixed methods research.

[5] Wiering B, de Boer D, Delnoij D (2017). Patient involvement in the development of patient-reported outcome measures: A scoping review. Health Expect.

[6] Special Section on Mixed Methods Research (2012). Qual Life Research.

[7] Roadmap to Patient-Focused Outcome Measurement in Clinical Trials: US Food and Drug Administration; 2015. Available from: https://www.fda.gov/Drugs/DevelopmentApprovalProcess/DrugDevelopmentToolsQualificationProgram/ucm370177.htm.

[8] Tashakkori A, Editorial CJW (2007). The new era of mixed methods. Journal of Mixed Methods Research.

[9] Busija L, Nicholson G, Toombs M, Cinelli R, Easton C, Sanders K, et al. Developing Conceptual Model of the Role of Elders in the Wellbeing of Australian Indigenous Communities: Group Concept Mapping Study 23rd ISOQOL Annual conference; Copenhagen, Denmark2016.

[10] Rosas SR, Ridings JW (2017). The use of concept mapping in measurement development and evaluation: Application and future directions. Eval Program Plann.

[11] Willgoss T. A Novel, Patient-Centered, Mixed Methods Approach to Identifying Relevant Concepts for Patient-Reported Outcome Measures and Facilitating Participant Engagement. 21st ISOQOL Annual Conference; Berlin, Germany2014.

[12] Barbic S. Development and testing of the Personal Recovery Outcome Measure (PROM) for people with mental illness: Application of Mixed methods. 23rd ISOQOL Annual conference; Copenhagen, Denmark2016.

[13] Blum SI, Bushnell DM, McCarrier KP, Martin ML, Cano S, Liedgens H, et al. The Pain Assessment for Lower Back Symptoms (PAL-S) and Impacts (PAL-I): A Case Example of the Application of Mixed-Methods in PRO Instrument Development. 23rd ISOQOL Annual Conference; Copenhagen, Denmark2016.

[14] Cleanthous S. Why we should move Mixed Methods in Psychometric Research from a three-step to a “two-step”. 23rd ISOQOL Annual Conference; Copenhagen, Denmark2016.

[15] Hudgens S. Application of mixed models for clinician reported outcome development 23rd ISOQOL annual conference; Copenhagen, Denmark2016.

[16] Fofana F, Bonnaud-Antignac A, Regnault A. A mixed method approach to help demonstrate saturation in qualitative research: applying Partial Least Square regression to qualitative data. 1st Mixed Methods International Research Association (MMIRA) Annual Conference; Boston, MA2014.

[17] Onwuegbuzie AJ, Bustamante RM, Nelson JA (2010). Mixed research as a tool for developing quantitative instruments. Journal of Mixed Methods Research..

[18] Gelhorn HL, Kulke MH, O'Dorisio T, Yang QM, Jackson J, Jackson S (2016). Patient-reported symptom experiences in patients with carcinoid syndrome after participation in a study of Telotristat Etiprate: A qualitative interview approach. Clin Ther.

[19] Marrel A, Fofana F, Guillemin I. Increasing the interpretability of patient-reported outcomes questionnaire findings using a mixed methods design: An example in a rare cardiac clinical trial. 19th ISPOR Annual European Congress; Vienna, Autria2016.

[20] Patient-Centered Outcome Measures Initiatives in the Field of Rare Diseases. International Rare Diseases Research Consortium; 2016.

[21] Kirwan JR, de Wit M, Frank L, Haywood KL, Salek S, Brace-McDonnell S (2017). Emerging guidelines for patient engagement in research. Value Health.

[22] Maxwell J, Mittapalli K, Tashakkori A, Teddlie C (2010). Realism as a stance for mixed methods research. SAGE handbook of mixed methods in social & behavioral research.

